# A molecular study on *Babesia* spp. in cattle and ticks in West-Azerbaijan province, Iran

**Published:** 2017-12-15

**Authors:** Sepideh Rajabi, Bijan Esmaeilnejad, Mousa Tavassoli

**Affiliations:** *Department of Pathobiology, Faculty of Veterinary Medicine, Urmia University, Urmia, Iran*

**Keywords:** *Babesia*, Cattle, Iran, Multiplex-PCR, Tick

## Abstract

A total number of 450 blood samples were collected from 45 different randomly selected cattle herds. Light microscopic examination of blood smears revealed *Babesia *spp. infection in 4.2%, while 8.9% of blood samples were positive using PCR. Upon multiplex-PCR (mPCR), *B. bigemina* and *B. bovis* infections were detected in 37/40 (92.5%) and 3/40 (7.5%) samples, respectively. 530 ticks of 10 Ixodid species were collected from the same cattle. *Hyalomma anatolicum* was the most prevalent tick species (19.9%). An expected 520 bp fragment of *Babesia *spp. was generated in 22 (48.8%) of *Rhpicephalus annulatus*, 18 (40.0%) of *R*. *bursa *and 12 (30.0%) *R. sanguineus sensu lato*. The mPCR findings revealed that all infected ticks including *R. annulatus*, *R*. *bursa *and *R*. *sanguineus* were totally infected with *B. bigemina*. The DNA amplification of *B. bovis* and *B. bigemina* in egg samples showed that only *B. bigemina* was detected in two specimens of *R. annulatus*. It could be concluded that *B. bigemina *was the dominant causative agent in this region but the evidence of *B. bovis *infection of cattle in a few cases was noted, as well. The results suggested that *B. bigemina *and *B. bovis* could be detected in the DNA extracted from *R. annulatus*, *R. bursa* and *R. sanguineus sensu lato *confirming previous reports. Since *B. bigemina* is transmitted transovarially by *R. annulatus*, it might act as an important vector for *B. bigemina*.

## Introduction

Bovine babesiosis, a tick-borne disease, is mainly caused by *Babesia bigemina* and *B. bovis* in the tropics and subtropics*.*^1^ Babesiosis caused by *B. bovis* has been reported to be more severe than that caused by *B. bigemina*.^1^ If animals recover from infection, a long-lasting carrier status occurs in which low numbers of erythrocytes remain infected with *Babesia* piroplasms. These carrier animals have an important role in the transmission of the infection by ticks.^1^ Therefore, early and correct diagnosis is essential to initiate proper treatment for the disease. 

Routine clinical diagnosis for babesiosis is usually based on the light microscopic detection of the piroplasms in Giemsa-stained blood smears and clinical signs in acute phase of the disease, but after acute infections, recovered animals frequently sustain subclinical infections, which is microscopically undetectable and lead to a relatively high rate of false negative diagnosis.^1^ Carrier animals play a critical role in epidemiology of *Babesia* since outbreaks may occur when carrier cattle, which have been incorrectly diagnosed as being clear of infection, are transported to new regions and serve as a reservoir of infection for naive cattle and ticks in non-endemic areas. It is also difficult to differentiate species of parasites on the basis of morphology. This makes the diagnosis problematic when the hemoparasites are often found together with in a single host.^2^ Serological diagnostic tests are frequently employed in determining subclinical infection. However, serology bears drawbacks such as cross-reactivity of antibodies between species and lacks sufficient sensitivity to detect infection in animals with low level of parasitemia.^2^ For this reason, the use of specific and sensitive molecular alternative techniques has become necessary for epidemiological investigations. Although individual polymerase chain reaction (PCR) assays based on the small subunit ribosomal RNA (SSU rRNA) gene designed to detect single species one at a time are effective, they can be time consuming and expensive when applied to a large number of samples that may be co-infected with a number of pathogen species. Furthermore, SSU rRNA gene is highly conserved, which restricts its use between closely related species.^3^ With these explanations, there is a need for a single, cost-effective and technically less demanding method that could specifically and differentially detect pathogens for diagnostic and epidemiological assessments of bovine babesiosis in endemic regions.^3^ Because internal transcribed spacers (ITSs) have great variability in nucleotide and length, ITS sequences were used for discriminating different geographic isolates of piroplasmids, identifying new species and differentiating between piroplasm species and subspecies. Therefore, multiplex-PCR (mPCR) based on ITSs offers a significant advantage over single-species detection systems for assessment of co-infection in a large number of samples.^3^ Regarding the bovine babesiosis importance as a lethal infection that imposed great constraints to livestock farming and also because of the paucity of data on prevalence of babesiosis among cattle in Iran, the present study as a first molecular diagnostic technique using mPCR was employed to detect and identify *Babesia *spp. in cattle and ticks in West-Azerbaijan province in northwest of Iran.

## Materials and Methods

**Fig. 1:**
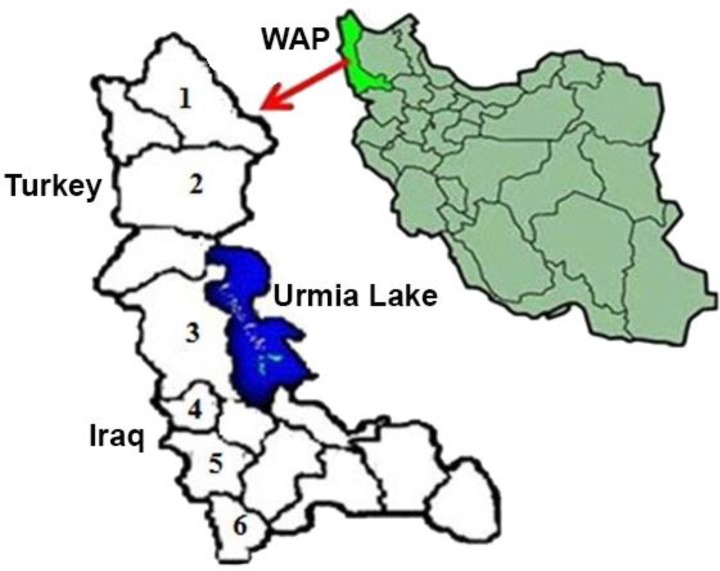
Map of West-Azerbaijan province (WAP), northwestern Iran, showing the locations surveyed in the current study. 1: Maku, 2: Khoy, 3: Urmia, 4: Oshnavieh, 5: Piranshahr and 6: Sardasht.

**Study area and sampling. **According to a 50.0% prevalence of babesiosis in cattle in the studied region, 5.0% absolute precision and 95.0.% confidence level, a total number of 450 cattle were sampled in the present study during favorable seasons, from early May through late September 2015. Cattles were randomly selected from 45 herds located in northern (Maku and Khoy, 17 herds), central (Urmia and Oshnavieh, 15 herds) and southern districts (Piranshahr and Sardasht, 13 herds) of West-Azerbaijan province in northwest of Iran (Fig. 1). From each herd, at least eight animals were randomly chosen. The examined cattle were raised under traditional husbandry practices (grazing on pastures during the day) without regular acaricide treatment. At sampling, data on the levels of the herd and animal were completed by the questionnaires. The herd-level variables included herd size (herds with 10 to 50 animals versus herds with more than 50 animals) and herd location (northern, central and southern areas). In each herd, cattle were categorized into two age classes (< 1 year old versus ≥ 1 year old). Herds were divided in two categories: herd with tick burden and no tick burden.

Jugular blood samples were collected into tubes containing ethylenediaminetetraacetic acid (EDTA) for DNA extraction and ear vein thin blood smears were immediately prepared and examined under an oil-immersion 1000× objective for the presence of intra-cellular forms of the parasite which morphologically were classified as *Babesia *spp. Parasitemia was expressed as the log number of red blood cells infected with *Babesia *parasites per 10^5 ^erythrocytes.^4^ The smears were recorded as negative for *Babesia *spp. if no parasites were detected in observed oil immersion fields. Ticks found on cattle at some of the survey sites were removed with rubbing alcohol pads surrounding the skin and blunt pointed forceps and then counted. Ticks were identified using the key identification guide.^5^

**Processing of ticks. **For demonstration of tran-sovarially transmission of *B.bigemina *and *B. bovis*, ten fully engorged female ticks from each species of ticks (a total number of 100 tick specimens) were individually placed on hollow plates and incubated at 27.0 ± 2.0 ˚C with 75.0 to 80.0% relative humidity for oviposition. On day 15 of oviposition, eggs masses laid were collected. Finally, the eggs and oviposition ticks were frozen at –70 ˚C for further use. The preparation of salivary glands was performed according to Estrada-Pena *et al*.^5^

**The DNA isolation.** The DNA was extracted from both blood and tick samples using a DNA isolation kit (MBST, Tehran, Iran), according to the manufacturer's instructions. Extraction of DNA from egg samples was performed according to the procedure described previously by Oliveira-Sequeira *et al*.^6^

**The PCR and mPCR reactions. **A pair of primers described by Georges *et al*.^7^ was used for generation approximately 460 bp and 520 bp fragments of *Theileria *and *Babesia *spp., respectively. For molecular identification of *Babesia *species, *Babesia *spp-positive blood was subjected to mPCR as described previously.^3 ^The extracted DNA from salivary glands of adult ticks and egg samples was amplified and then differentiated according to the protocol previously described for blood samples. The positive control for *Babesia *(accession numbers EF547924 and EF547925) was provided from cattle with clinical babesiosis (diagnosis was done based on clinical signs and light microscopic examination Giemsa stained thin blood smear) by Pasteur institute in Iran. Distilled water was served as a negative control. Finally, PCR and mPCR products were electrophoresed and visualized under UV transilluminator (20M; BTS, Tokyo, Japan). 

**Statistical analysis** ***.*** The Fisher’s exact test was used to express association between the presence (positive and negative blood samples) of *Babesia* and the various parameters, i.e. herd size, gender and age of animal, tick infestation of cattle and presence of ticks in the herd. The SPSS software version 22.0 was used to compare the data of blood smears with blood PCR method. Results were displayed as *p* values as well as relative risk values (with 95 % confidence intervals). A *p *value less than 0.05 was accepted to be statistically significant.

## Results

**Fig. 2:**
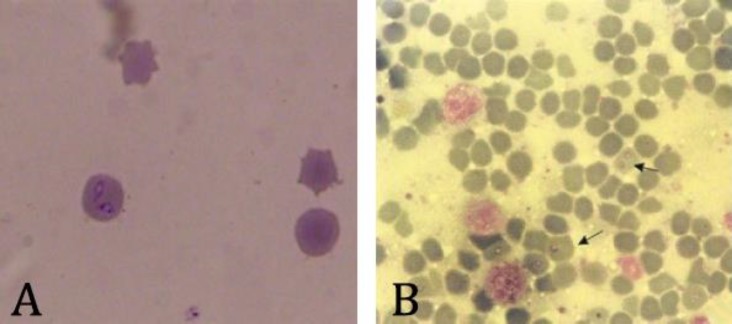
*Babesia *spp. inside cattle red blood cell. (A) *B. bigemina*; (B) *B. bovis *(Giemsa, 100×).

**Examination of blood smears. **The results showed that 19 (4.2%) animals were positive for *Babesia *spp*.* upon microscopic examination, of which 1 animal (0.2%) and 18 animals (4.0%) were morphologically compatible with *B.bovis* and *B. bigemina*, respectively. They were appeared as circular, oval and pear-shaped bodies within red blood cells (Fig. 2). 

**Tick infestation** ***.*** In this study 530 ticks specimens were collected from 450 animals thus mean intensity for each animal was 1.18. As Table 1 shows, the highest tick number was distributed in the northern areas (48.6%) and lowest number in central areas (18.0%). The most of ticks were found on the host during July (57.9%), but a few collections were found on September (2.8%).

 A total number of 10 species of Ixodid ticks including *Hyalomma anatolicum *19.9%, *H*. *asiaticum *18%, *H*. *excavatum *12.4%, *H*. *detritum *9.8%, *R. annulatus *8.4%, *R. sanguineus sensu lato *7.6%, *R*. *bursa *7.6%, *R. turanicus *6.0%*, Dermacentor marginatus *5.8% and *Haemaphysalis punctata *5.0% were isolated from infested cattle in West-Azerbaijan province, Iran (Table 2).

**Fig. 3:**
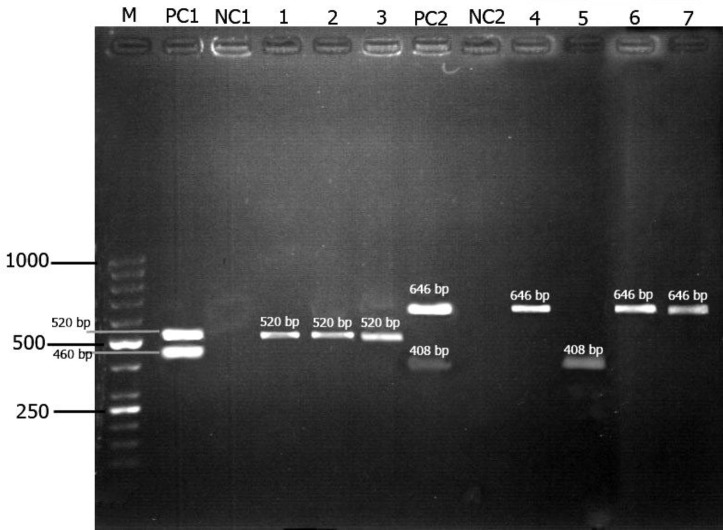
Simple PCR (A) and mPCR (B) for detection of *B. bigemina *and *B. bovis*. Lane M: Molecular marker (50bp ladder); Lane PC1: *Theileria *and *Babesia *spp. positive control; NC1, NC2: Negative control; Lane 1: *Babesia* spp- infected blood; Lane 2: *Babesia* spp- infected tick; Lane 3: *Babesia* spp- infected egg; Lane PC2: *B. bigemina *and *B. bovis* positive control; Lane 4: *B. bigemina*-posive blood; Lane 5: *B. bovis*-positive blood; Lane 6: *B. bigemina*-infected ticks; Lane 7: *B. bigemina*-infected egg.

**Detection and differentiation of** ***Babesia*** **spp.** The prevalence of babesiosis in cattle detected by PCR was significantly higher than those obtained by microscopic examination of their blood smears (*p *< 0.05). The prevalence of *Babesia* infection in age groups and different gender were significantly different (*p *< 0.05). Frequency of *Babesia *infection was significantly higher in herds with tick burden than no tick burden (*p *< 0.05, Table 3). The relative risk with 95% confidence intervals was 2.9 for tick burden of animal. *Babesia* *bigemina* was the most prevalent (37/40), compared to *B. bovis* (3/40), (Fig. 3). Therefore, the prevalence of *B. bigemina* (92.5%) in these areas was significantly higher (*p *< 0.05) than *B. bovis* (7.5%), (Table 4).

The examination of 530 ticks revealed that an expected 520 bp fragment of *Babesia *spp. was generated in 22 cases (48.8%) of *R. annulatus*, 18 (40.0%) of *R*. *bursa *and 12 (30.0%) of *R. sanguineus sensu lato *(Table 3). The difference of positive rate for male and female ticks was statistically different (*p *< 0.05, Table 2). The monthly related prevalence of infection in cattle, *R. annulatus*, *R. bursa* and *R. sanguineus sensu lato *was the highest in July, while a decrease was observed in September (*p *< 0.05).

The results of DNA amplification of *B. bovis* and *B. bigemina* in egg samples showed that two ticks of 100 (6.7%) engorged female ticks yielded a specific *Babesia* fragment (520 bp). Multiplex PCR revealed that only *B. bigemina* was detected in two specimens of *R. annulatus*.

**Table 1:** The distribution of tick species (including *Hyalomma *and *Rhpicephalus*) infested cattle during tick active season in West-Azerbaijan province, Iran.

**Area and month**	***H. anatolicum***	***H. siaticum ***	***H. excaraticum***	***H. detritum***	***R. annulatus***	***R. bursa***	***R. sanguineus ***	***R. turanicus***	***D. marginatus***	***H. punctata***	**Total (%)**
**Northern**	35	40	30	12	25	25	20	25	20	26	258 (48.6)
**Central**	40	25	-	30	-	-	-	-	-	-	95 (18.0)
**Southern**	30	39	36	10	20	20	15	7	9	-	177 (33.4)
**Total**	105	104	66	52	45	45	35	32	29	26	530
**May**	7	4	5	2	6	3	5	-	2	3	37(6.6)
**June**	16	27	10	8	7	6	11	5	4	7	100 (18.9)
**July**	47	45	46	40	25	24	20	23	20	14	307 (57.9)
**August**	32	16	4	2	4	4	3	4	2	2	73 (13.8)
**September**	3	3	1	-	3	3	1	-	1	-	15 (2.8)
**Total**	105	95	66	52	45	40	40	32	29	26	530

**Table 2:** The infection frequency of tick species (including *Hyalomma *and *Rhpicephalus*) infested cattle and percentage of infection with *Babesia *spp. in West-Azerbaijan province, Iran. Data within the parentheses are the percentage of infestation.

**Tick species**	**Tick number **	**Male**	**Female**	**Total infected ticks with ** ***Babesia*** ** spp.**				**Male tick infected with ** ***B. bigemina***				**Female ticks infected with** ***B. bigemina***			
				**N**	**C**	**S**	**Total**	**N**	**C**	**S**	**Total **	**N**	**C**	**S**	**Total**
***H. antolicum***	105(19.9)	60	51	-	-	-	-	-	-	-	-	-	-	-	-
***H. asiaticum***	95(18.0)	54	41	-	-	-	-	-	-	-	-	-	-	-	-
***H. excaratum***	66(12.4)	48	12	-	-	-	-	-	-	-	-	-	-	-	-
***H. detritum***	52(9.8)	28	24	-	-	-	-	-	-	-	-	-	-	-	-
***R. annulatus***	45(8.4)	35	10	14(31.1)	2(44.0)	6(13.3)	22(48.8)	2(4.4)	-	1(2.2)	3(6.6)	12(26.7)	2(4.4)	5(11.1)	19(42.2)
***R. bursa***	40(7.6)	25	15	12(30.0)	1(2.5)	5(12.5)	18(40.0)	1(2.5)	1(2.5)	1(2.5)	3(7.5)	11(27.5)	-	4(10.0)	15(37.5)
***R. sanguineus ***	40(7.6)	30	10	10(25.0)	-	2 (5.0)	12(30.0)	-	-	1(2.5)	1(2.5)	10(25.0)	-	1(2.5)	11(27.5)
***R. turanicus***	32(6.0)	15	17	-	-	-	-	-	-	-	-	-	-	-	-
***D. marginatus***	29(5.8)	15	14	-	-	-	-	-	-	-	-	-	-	-	-
***H. punctata***	26(5.0)	10	16	-	-	-	-	-	-	-	-	-	-	-	-
**Total**	530	320(60.3)	210(39.71)	36(6.7)	3(0.5)	13(2.4)	52(9.7)	3(0.5)	1(0.1)	3(0.5)	7(1.3)	33(6.2)	2(0.3)	10(1.9)	45(8.4)

N = Northern, C = Central, S = Southern.

**Table 3:** Association between the presence (PCR-positive and negative blood samples) of *Babesia* infection in cattle and the studied parameters describing animal and herd characteristics. Data within the parentheses are the percentage of infestation.

**Total of cattle**		**Herd size**		**Herd location**			**Age of animal**		**Gender of animal**		**Tick burden **	
		**10-50 animals**	**> 50 animals**	**N**	**C**	**S**	**< 1yr**	**≥ 1yr**	**Male**	**Female**	**No tick**	**> 1**
**Number**	450	280	170	150	150	150	162	288	218	232	310	140
**Negative**	410(91.1)	264(94.2)	156(91.8)	135(90.0)	138(92.0)	137(91.3)	153(94.5)	275(89.2)	208(95.4)	202(87.1)	297(95.9)	55(80.8)
**Positive**	40(8.9)	16(5.8)	14(8.2)	15(10.0)	12(8.0)	13(8.7)	9(5.5)	31(10.7)	10(4.6)	30(12.9)	13(4.1)	27(19.2)
***p*** **(F)**		0.06 (NS)		0.52 (NS)			0.04		0.03		0.002	

*p*(F): *p* value of Fisher's exact test; N: Northern, C: Central, S: Southern; NS: Not significant.

**Table 4:** Results of microscopic examination, PCR and mPCR for *Babesia* spp. in different areas of the West-Azerbaijan province, Iran. Data within the parentheses are the percentage of infestation.

**Area**	**Number of ** **sample**	**Microscopic observation**				**PCR**		**mPCR**		
		**Positive sample**		**Parasitemia**		***B. bigemina***		***B. bovis***	**Concurrent**	
		***B. bigemina***	***B. bovis***	***B. bigemina***	***B. bavis***	**positive**	**positive**	**positive**	**positive**	**%**
**Northern**	150	7 (4.7)	-	9.0%	-	15(10.0)	14(93.4)	1(13.3)	-	-
**Central**	150	6 (4.0)	-	2.0%	-	12(8.0)	11(91.7)	1(8.3)	-	-
**Southern**	150	5 (3.4)	1 (0.7)	14.0%	0.2%	13(8.7)	12(92.3)	1(7.6)	-	-
**Total**	450	18 (4.0)	1(0.2)	8.4%	0.2%	40(8.9)	37(92.5)	3(7.5)	-	-

## Discussion

Tick-borne diseases (TBD) pose major problems for the health and management of domestic cattle in Iran.^1^ Among these diseases, bovine babesiosis is the most prevalent and economically important.^4^^,^^6^ Also, the laboratory diagnosis of babesiosis in many parts of Iran is done by a combination of clinical findings and examination of stained smears of peripheral blood as well as serological investigations. However, these methods are not reliable and efficient enough to study on the epidemiological aspects of bovine babesiosis. On the other hand, taking into account the limitation of serological and PCR-associated methods, e.g. reverse line blot and nested-PCR,^3^ in this study mPCR-based molecular detection and identification of *babesia *spp. in cattle and ticks were performed in West-Azerbaijan province in northwest of Iran.

In the present study, *Babesia* spp. infection was observed in 4.2% of blood smears of the cattle in West-Azerbaijan province. Our results were closest to the results of Fakhar *et al*.^8^ and Noaman^9^ from Iran and Ekici and Sevinc^10^ from Turkey, who reported that prevalence of *Babesia* spp. was varied from 0.0 to 2.1% in Iran and 1.4 to 4.2% in Turkey, respectively. On the other hand, the frequency rates of *Babesia *spp. infection were reported from 7.1 to 18.1% in different areas of Iran using light microscopy.^11^^,^^12^ Also, prevalence of *Babesia* spp. in cattle was detected microscopically in other countries of Middle East such as Iraq (9.0%)^13^ and Turkey ranged from 11.4 to 62.0%.^14^^,^^15^ These dissimilar results could probably be ascribed to that the blood samples of present study were taken from every cattle and might show a lower frequency than other studies that were sampled from cases with a known history of disease. 

Microscopically, *B. bovis* and *B. bigemina *were detected among cattle in the studied area with prevalence of 0.2% and 4.0%, respectively. *Babesia bigemina *had been previously reported to occur in Iran with 2.1% prevalence rate,^8^ but there appears to be no report about *B. bovis *infection in cattle in Iran to compare with the present study. However, Aktas and Ozubek reported 9.0% infection of cattle by *B. bovis *in Turkey.^16^ The different results of two studies may be related to that the blood samples in latter study were taken from cattle exhibiting recumbency, ataxia, incoordination and mild anemia.

 In the microscopic examination it was found that parasitemia was ranged from 0.04 to 0.2% for *B. bovis* and 2.0 to 14.0% for *B. bigemina*. The highest parasitemia seen in southern areas is likely due to that these areas are located near Iraq border where climate conditions that affect the intensity of ticks that feeding on hosts are effective on parasitemia ratio and severity of disease. Our results concerning low parasitemia in *B. bovis *infections compared to *B. bigemina* weresupported the view that in contrast to *B. bovis* infection, which was characterized by low level of peripheral parasitemia (up to 0.2%), parasitemia may be more than 40.0% of erythrocytes in *B. bigemina*-infected cattle.^16^^-^^18^ Low parasitemia rates of *B. bovis *most probably be due to the ability of parasitized erythrocytes to sequester by *B. bovis*-glycosyl-phosphatidylinositol-anchored protein in microcapillaries of the kidneys, lungs and brain.^19^

 In a previous study in Turkey, serological tests employing immuno-fluorescence assay were used and the seropositivity rate of *B. bigemina *in cattle was varied from 33.5% to 54.8% in different regions of the country.^20^^,^^21^ Also, Ameen *et al*., using enzyme-linked immunosorbent assay, reported 27.2% infection of cattle by *B. bigemina *in Iraq.^22^ In the present study, covering West-Azerbaijan province in north-west, Iran, the prevalence was ranged from 8.0 to 10.0% on the examined farms. Although the results of the present and previous studies cannot be compared due to the different methods employed, the results clearly indicated that *Babesia* was broadly dispersed in north-west of Iran and neighboring countries.

In the present study, the prevalence of *Babesia *infection in cattle detected by PCR (8.9%) was significantly higher than one diagnosed through microscopic examination of thin blood smears (4.0%). Therefore, DNA amplification methods had higher efficiency than microscopic examination for detection of *Babesia*. The results were in agreement with a previous report about bovine and ovine babesiosis.^4^^,^^23^

According to our results, the infection rate of bovine babesiosis was significantly higher in aged cattle. The results also confirmed the results of other investigations according to the age-related immunity to babesiosis.^1^^,^^24^ In previous observation there was no difference between *Babesia* spp. prevalence in all ages and significant higher *Babesia* spp. prevalence in young cattle.^25^^,^^26^ In general, young and adults are susceptible to babesiosis, while in young cattle maternal antibodies persist for the longer period of three months.^27^ Also, there are no data to explain these results, the presence of fetal haemoglobin (HbF) in the calves could represent a possibility since HbF is considered as one of the factors contributing to the high resistance of young cattle against *Babesia* infection.^6^ Concerning sex susceptibility to infection, current study showed higher rate of infection in female cattle. The physiology of the female during pregnancy and lactation period which is associated with hormonal and immunological changes could explain this finding.^28^

The finding that the prevalence of bovine babesiosis was higher in herds with tick burden indicates the presence of a positive correlation between the prevalence of the disease and the presence of vector ticks. This finding was consistent with the findings of Esmaeilnejad *et al*.^4^ and Theodoropoulos *et al*.^29^

Based on previous studies, *B. bigemina* was reported as the most prevalent and main causative agent of bovine babesiosis in Iraq,^22^ Egypt,^30^ India^31^ and Turkey^14^^,^^32^^-^^34^ however, in a recent study carried out in Black Sea region of Turkey; *B. bovis* was found as a predominant *Babesia* species in cattle.^16^ This contradiction might be because of: first, latter study carried out in small sample size (small-scale) and second, our collected ticks were all obtained from semi-arid zone, while the ticks collected by Aktas and Ozubek,^16^ were from the Black Sea region of Turkey where occupies a coastal area with a humid oceanic climate. This bioclimatic condition provides suitable habitat for *Ixodes ricinus* that is the main transmitter agent for *B. bovis*.^35^

 In our study, 10 species of ticks were identified in cattle herds in West-Azerbaijan province, Iran in which, *H. anatolicum *was the most frequent and abundant tick species. Fauna diversity and different frequency of ticks observed in the present study were in agreement with those reported earlier from the western half of Iran^36^^-^^38^ as well as eastern of Iraq^39^ and Turkey.^20^^,^^40^^,^^41^ In disagreement with these findings, Riabi and Atarodi^42^ and Gharekhani *et al*.^43^ previously reported that *H*. *excavatum* infestation was more frequent than *H.anatolicum *in cattle in south Khorasan-e-Razavi and Hamedan provinces. A geographical disparity between two regions may have resulted to better adaptation of one tick’s species to the local conditions, thus replacing with other ones.

Based on mPCR results, *B. bigemia *was detected in *R. annulatus*, *R. bursa* and *R. sanguineus sensu lato*. These results were consistent with the findings of other researchers.^43^^,^^44^ The results were slightly different from those obtained by Tavassoli *et al*.^36^ who found that *Babesia* infection was detected in all of *Rhipicephalus *spp. except *R. annulatus*. It seems that low number of *R. annulatus* samples (n = 6) in Tavassoli and colleagues’ survey may account for this difference. 

In the present study, the prevalence rate of *Babesia *infection was significantly higher in female ticks than males. It was reported that female ticks had many more type III acini than male ticks; therefore, the prevalence and intensity of *Babesia *infection were significantly higher in female ticks than males.^39^ On the other hand, the fact that because *Babesia* parasite can infect the ovaries and be transmitted transovarially via the eggs, so that all stages of female ticks are potentially infective.^45^ These statements show why female ticks have greater *Babesia *infection prevalence than males. The results of the present study agree with those of the above-mentioned researchers.

 Season, climate and soil type regulate tick population and its geographical distribution. The peak activity of *Rhipicephalus* species was mostly occurred during spring and summer (April to August) in steppe climate areas on northern mountain slopes covered with low vegetation. Also, adult ticks become active in the field with abundant livestock hosts when average annual precipitation is between 300 to 600 isohyets (more than 600 mm).^46 ^The bio-ecologic features of West-Azerbaijan province show that, as the green plants or year-long vegetation increase from northern areas to southern areas, precipitation and population of livestock hosts are decreased. Climatic structure would thus permit development of ticks in northern areas of West-Azerbaijan province and subsequently facilitate the long-term persistence of *Babesia* species.

In this study, the highest prevalence of *Babesia* infection was observed in July (16.1%), corresponding to the most active adult vector ticks. Other studies have shown that there is a close relationship between rise in *Babesia* spp. infection and seasonal activity of vector ticks.^47^^-^^49^

According to the present study, vertical transmission for *B. bigemina *has exclusively been demonstrated in *R. annulatus*. The results were in agreement with previously reported findings.^49^ Low possibility of transovarian transmission of *Babesia *could be attributed to: a) *B. bigemina* and *B. bovis* transmitted transovarially by one-host *Rhipicephalus* spp. ticks; thus, among *Rhipicephalus* spp. ticks only *R. annulatus *has this ability and b) vertical infection does not occur in *B. bovis *due to longicine and longipain, two antimicrobial peptides produced in the *Rhipicephalus *mid gut epithelium, which inhibit proliferation and kill *babesia *merozoites^50^ and although possible for *B. bigemina*, it is much less efficient because of additional kinete detected in female hemolymph causes decreases in the egg and larval hatchability.^25^

In conclusion, *B. bigemina *was the dominant causative agent in this region but the evidence of *B. bovis *infection of cattle in a few cases was noted, as well. *R. annulatus*, *R. bursa* and *R. sanguineus sensu lato *could transmit *B. bigemina* and *B. bovis* to cattle. Because only *B. bigemina* is transmitted transovarially by *R. annulatus*, it may act as an important vector for *B. bigemina*. Sequencing of mPCR products will clarify more detailed information about molecular characterization and genetic heterogeneity of bovine *Babesia* spp. In addition, experimental studies are recommended to determine whether *B. bovis *could be transmitted by *Rhipicephalus* spp.
